# Volume Sixth

**Published:** 1889-03

**Authors:** 


### Editorial.

### VOLUME SIXTH.

In commencing a new volume with the present number, the JOURNAL takes pleasure in announcing to its readers that it has secured the services of Dr. Virgil O. Hardon, of Atlanta, who will henceforth assume the active editorial management. It is believed that this change will be conducive to the highest interests of the JOURNAL and its readers. Dr. Hardon brings to this work an experience gained by a long connection with one of the best medical periodicals at the North. During the past three years he has been a frequent contributor to this JOURNAL, and our readers have had ample opportunity to judge of his merits as a medical writer.

Drs. W. F. Westmoreland and H. V. M. Miller will still retain their connection with the editorial staff, as heretofore, and the influence and efforts of these veterans of medical journalism will continue to be exerted in keeping the JOURNAL up to the high standard of excellence which it has maintained in past years.

It will be the policy of the JOURNAL to devote the larger part of its space to well selected original contributions from the best writers in this country. From time to time we shall present editorial translations of short practical articles of value from the latest French and German sources, thus enabling our readers to keep pace with the advances in medicine abroad as well as at home. Preference will be given to matter which has practical value to the busy practitioner and will aid him in his daily calling. Yet the fact will not be lost sight of that medicine is a science, as well as an art, and that a successful practice must have its foundation in an accurate scientific knowledge of the causation and pathology of disease.

The editorial department will be given greater prominence than before, and live questions of the day will be fearlessly and impartially discussed. The other features of the JOURNAL, which have heretofore added to its value and interest, will be continued unchanged.

If earnest, conscientious endeavor can accomplish that at which it aims, the JOURNAL will be better this year than ever before. Profiting by the experience of the past and availing itself of increased facilities and opportunities, it enters upon this new volume in the hope that the reputation already attained by it as the leading medical journal in the South, will in nowise suffer in the hands of the new editorial management.

### ITEMS.

Is your life insured ?

WHAT is to become of all the young doctors, who, in the next thirty days, will be cast upon this cold, cold world?

THE JOURNAL will be better than ever this year. Now is the time to subscribe.

THE Medical Association of Georgia will meet in Macon April 17th, 1889.

THE Medical Association of the State of Alabama will meet in Mobile Tuesday, April 9th, 1889.

Now is the time to prepare your paper for the State Society meetings.

DR. JNO. C. DALTON, the eminent physiologist of New York, died February 12th, 1889.

DR. R. H. DAY, of Baton Rouge, La., one of the most distinguished physicians of that State, has accepted the chair of Diseases of Children in the New Orleans Polyclinic.

It is said that the city council of Jacksonville has employed E. W. Bowditch, sanitary expert of Boston, to make a thorough examination of the sewerage system of Jacksonville.

"Methylbenzoinethoxyethyltetrahydropyridinecasboxylate," is the chemical terminology for cocaine. No wonder students fail to see the beauties of chemistry.

A NEW WAY OF DIAGNOSTICATING SHOULDER PRESENTATION.—Quiz Master—How do you diagnosticate a shoulder presentation? Student—By feeling for the hair in the axilla.—*Medical Record.*

THE thirty-first annual commencement exercises of the Atlanta Medical College will occur on Monday night, March 4th, 1889. The session has been an unusually prosperous one. Catalogues for the next course will be issued about July 1st.

The hospital of the Johns Hopkins University will be formally opened May 1. Its organization has been entrusted to President Gilman, who, it is said, will reside in the hospital and exercise a close personal supervision over its executive management.

The Supreme Court of the United States has rendered a decision in regard to the validity of the West Virginia Medical Practice Act, that will set at rest a great deal of the discussion as to the constitutionality of Medical Practice acts. The following is a part of the decision:

"If the means adopted are appropriate to the calling or profession, and obtainable by reasonable study and application, no objection to their validity can be raised."

A BIBLICAL ARGUMENT AGAINST ANÆSTHESIA during parturition made by a pompous priest in France, was recently answered by another, made by a woman who had "been there"—which the priest had not, at least in that way. A lady, very much perturbed in mind concerning the ordeal through which she was destined soon to pass, addressed a letter to Msgr. Hugues Le Roux, asking whether she had the right (spiritual) to allow herself to be anæsthetized during parturition. His answer was, "You have not the right to place in peril your own life and that of the child to be born of you, simply to avoid the pangs of parturition. If a physician worthy of confidence is willing to accept these risks, act according to the dictates of your faith and your scruples. Remember the text: 'I will multiply thy sorrow and thy conception; in sorrow thou shalt bring forth children.' Child bearing is a moral act as well as a physical phenomenon. The griefs of soul consequent upon maternity do not cease with the birth of the child, and for them medicine will never find a remedy." This being published in *Le Temps*, of which M. Hugues Le Roux is the *chronicleur spirituel*, the lady above alluded to writes a reply, which if not so full of piety is loaded with wit and good sense. "Now," says she, "do yo want *my* opinion ? You are mighty glib with your biblical verses which condemn *us* poor creatures to bear the pangs of child-bearing without seeking for relief. There was but one man ever confined, and he but once in his life—and he was put to sleep! Re-open your Scriptures, I pray you, and you will there find that when Adam was about to be delivered of Eve, God himself executed the operation and placed our ancestor in a deep sleep as a preliminary." Since the Bible is be appealed to in matters of this sort, it is exceedingly fortunate that it can be quoted on both sides of almost any question.

